# The Impact of Psychological Distress on Incident Functional Disability in Elderly Japanese: The Ohsaki Cohort 2006 Study

**DOI:** 10.3390/ijerph15112502

**Published:** 2018-11-08

**Authors:** Yasutake Tomata, Takashi Watanabe, Fumiya Tanji, Shu Zhang, Yumi Sugawara, Ichiro Tsuji

**Affiliations:** Division of Epidemiology, Department of Health Informatics and Public Health, Tohoku University School of Public Health, Graduate School of Medicine 2-1, Seiryo-machi, Aoba-ku, Sendai 980-8575, Japan; tkswatanabe@med.tohoku.ac.jp (T.W.); ftanji-thk@umin.ac.jp (F.T.); zhangshu@med.tohoku.ac.jp (S.Z.); yumi1717@med.tohoku.ac.jp (Y.S.); tsuji1@med.tohoku.ac.jp (I.T.)

**Keywords:** psychological distress, K6, disability, elderly, population attributable fraction

## Abstract

*Background:* Although psychological distress is known to be a risk factor for death, there are relatively few data on the impact of psychological distress on incident functional disability in older adults. The aim of this study was to examine the impact of psychological distress on incident functional disability in older adults. *Methods:* We conducted a cohort study of 12,365 disability-free individuals aged ≥65 years who live in Ohsaki City, Japan. In 2006, the level of psychological distress was assessed using the K6 (range: 0–24 points). Data on 10-year functional disability were retrieved from the public Long-term Care Insurance database. The multivariate-adjusted hazard ratios (HRs) and population attributable fractions (PAFs) according to the K6 groups (<5, 5–9, 10–12, and ≥13 points) were estimated. *Results:* Among 94,636 person-years, incident functional disability occurred in 4533 persons (36.7%). Significantly higher risk was observed in higher K6 score groups. The multiple-adjusted HRs (95% CIs) of incident functional disability were 1.14 (1.06–1.22) for 5–9 points, 1.28 (1.15–1.43) for 10–12 points, and 1.62 (1.44–1.84) for ≥13 points, in comparison with <5 points (*p*-trend < 0.001). The PAFs in each of the K6 score groups were 3.0% for 5–9 points, 1.7% for 10–12 points, and 2.6% for ≥13 points. *Conclusions:* Even when mild to moderate, psychological distress had a considerable impact on incident functional disability in this cohort.

## 1. Introduction

Prevention of functional disability among the older people is a significant healthcare issue [1]. The risk factors for functional disability have been studied in terms of not only physical status but also psychological factors [2,3]. Depression is well known as to be a risk factor for functional outcome [2,3,4,5], but major depression is relatively rare among the older population (e.g., weighted average prevalence 1.8% in USA, 0.4% in Japan [6]).

Psychological distress would be a more practical concept in the context of comprehensive mental disorder when considering public health strategies [7]. Psychological distress is suggested as a risk factor for mortality [8,9]. Although relatively few data are available, several studies have reported that psychological distress is risk factor for incident disability [10,11,12]. A cross-sectional study investigating the impact (population-attributable risk) of psychological distress concluded that it is the most important factor that limits activity in older adults [12]. Psychological distress is indeed prevalent among older adults (prevalence of K6 score ≥5 points among Japanese adults aged ≥65 years is 22.7% [K6 score of 5–12: 20.48%, K6 ≥ 13: 2.25%]) [13], and therefore may impact considerably on incident functional disability in that age group.

To verify this hypothesis, a prospective study to investigate the impact would be needed, because a cross-sectional study would generally be limited by reverse causality. To our knowledge, however, no prospective study has investigated this issue especially with regard to the impact of several levels of psychological distress.

The aim of the present study was to examine the impact of psychological distress on incident functional disability in older adults, focusing particularly on population attributable fractions (PAFs) calculated for several levels of psychological distress.

## 2. Materials and Methods

### 2.1. Study Cohort

The design of the Ohsaki Cohort 2006 Study has been reported in our design paper [14]. In brief, the source population for the baseline survey comprised all older citizens resident (31,694 men and women aged ≥65 years) in Ohsaki City, Miyagi Prefecture, northeastern Japan, on December 2006. The survey included questions about psychological distress assessed by the Kessler six-item psychological distress scale (K6), as well as items on history of disease, motor function score, smoking, alcohol drinking, body weight, height, and social support.

The baseline survey was conducted between 1 and 15 December 2006, and the follow-up survey between 16 December 2006 and 30 November 2016. A questionnaire was distributed by the heads of individual administrative districts, and then collected by mail. In this survey, 23,091 persons who provided valid responses formed the study cohort. We excluded 6333 persons who did not provide written consent for review of their Long-term Care Insurance (LTCI) information, 1979 persons who had already been certified as having a disability by the LTCI at the time of the baseline survey, five persons who had died or moved away during the period of the baseline survey, and 2409 persons who left blank responses on the K6. Thus, 12,365 persons were analyzed for the purpose of this study.

During the 10-year study follow-up period, only 173 persons were lost to follow-up because of emigration from the study area; the follow-up rate was 98.6%. From the resulting 94,636 person-years, the number of persons who developed incident functional disability was 4533 (36.7%).

### 2.2. Psychological Distress

The exposure was psychological distress, as assessed by the K6 [15]. The Japanese version of the K6 has been validated in a previous study [16]. The K6 consists of six questions about how often an individual has felt the following in the last month: (1) nervous; (2) hopeless; (3) restless or fidgety; (4) so sad that nothing could cheer you up; (5) everything was an effort; and (6) worthless. The total K6 score ranged from 0 to 24. In previous studies, three cut-off points of 5, 10 and 13 have been commonly used to screen for psychological stress [10,17,18,19,20,21,22]. Thus, we classified participants into four groups according to their K6 score (<5, 5–9, 10–12, and ≥13 points).

### 2.3. Covariates

History of disease was collected by the question which asked whether participants have history about each disease (stroke, hypertension, myocardial infarction, diabetes, arthritis, and cancer).

Motor function was assessed by the Kihon Checklist including five binary questions (total point scores ranging from 0 to 5). We classified individuals with scores of <3 as having better motor function according to the validation study [23].

Smoking and alcohol drinking were assessed using questions that required a response of never, former, or current.

Self-reported weight and height were used to calculate BMI as weight (kg) divided by the square of height (m).

The degree of social support was assessed by asking the following five binary questions [24]: (1) Do you have someone with whom you can talk when you are in trouble? (2) Do you have someone whom you can consult when you do not feel well? (3) Do you have someone who can help you with your daily housework? (4) Do you have someone who can take you to a hospital when you feel ill? and (5) Do you have someone who can take care of you if you become bedridden? This questionnaire was available only in Japanese. A validation study has reported that persons who responded “yes” had a higher mean score on the Lubben Social Network Scale [25]. In the previous study, if subjects considered themselves to have all five forms of support, they were defined as having “no lack” of social support, as detailed in previous studies [26,27,28].

### 2.4. Follow-Up (Incident Functional Disability)

Incident functional disability was defined by disability certification of the LTCI system. The LTCI is a mandatory social insurance to assist daily activities of the disabled elderly [29,30,31]. Every person aged ≥65 years is eligible for formal caregiving services. To receive caregiving services through the LTCI system, a person must be certified according to the nationally uniform standard. The procedure for disability certification comprises two parts: (1) assessment of the degree of functional disability using a questionnaire developed by the Ministry of Health, Labor, and Welfare; and (2) reference to the Doctor’s Opinion Paper prepared by the attending physician. If a person is judged to be eligible, the Municipal Certification Committee decides on one of seven levels, ranging from Support Level 1, Support Level 2, and Care Level 1 to Care Level 5. A previous study has shown that the level of LTCI certification is well correlated with ability of ADLs and with the Mini-Mental State Examination score [32]. LTCI certification has already been used as an outcome measure of incident functional disability in elderly individuals [26,27,28,33,34]. We also included mortality as an end point, certified by the local registry.

We obtained a dataset that included information on LTCI certification, death, or emigration from Ohsaki City based on an agreement with Ohsaki City Government related to Epidemiologic Research and Privacy Protection.

### 2.5. Ethical Issues

We considered the return of completed questionnaires to imply consent to participate in the study involving the baseline survey data and subsequent follow-up of death and emigration. We also confirmed information regarding LTCI certification status after obtaining written consent along with the questionnaires returned from the subjects at the time of the baseline survey. The Ethics Committee of Tohoku University Graduate School of Medicine (Sendai, Japan) reviewed and approved the study protocol.

### 2.6. Statistical Analysis

We counted the person-years of follow-up for each subject from 16 December 2006 until the date of incident functional disability, date of emigration from Ohsaki City, date of death, or the end of the study period (30 November 2016), whichever occurred first.

We used the Cox proportional hazard model to calculate the hazard ratios (HRs) and 95% confidence intervals (CIs) for incident functional disability according to the K6 score groups. Dummy variables were created for the K6 score groups, and the group with the lowest K6 score was used as a reference category. Multivariate models were adjusted for the following variables. Model 1 was adjusted for sex and age (65–69, 70–74, 75–79, 80–84, or ≥85 years). Furthermore, Model 2 was adjusted for history of disease (stroke, hypertension, myocardial infarction, diabetes, arthritis, and cancer) (yes, no), motor function score (<3, ≥3, or missing), smoking (never, former, current, or missing), alcohol drinking (never, former, current, or missing), body mass index (in kg/m^2^; <18.5, 18.5–24.9, ≥25.0 or missing), and social support (lacking, no lack, or missing).

To consider the influence of excluding 2409 persons who left blank responses on the K6, we also checked the multiple-adjusted HRs (95% CIs) using analysis by the multiple imputation method (*n* = 14,774) employing IBM SPSS Statistics ver. 24 (IBM Software Group, Chicago, IL, USA). We generated 10 different missing datasets.

We also calculated the PAFs for incident functional disability in the various K6 score groups. Two PAFs were estimated for the K6 score groups: (1) PAF_ALL_ assuming that the K6 score for all subjects would have changed to the lowest level (<5 points); and (2) PAF_+1_ assuming that the K6 score for every subject would have improved by one level (e.g., a change from ≥13 points to 10–12 points), except for subjects with the lowest K6 score (<5 points). All PAFs and their 95% CIs were estimated using the “punafcc package” of Stata/MP 14 (StataCorp., College Station, TX, USA) [35]. Adjustment items for the PAFs were the same as those in Cox Model 2 mentioned above.

All data were analyzed using SAS version 9.4 (SAS Inc., Cary, NC, USA) except for the PAFs and the multiple imputation method. All statistical tests described here were two-sided, and differences at *p* < 0.05 were accepted as significant.

## 3. Results

### 3.1. Characteristics of the Subjects

The mean age (SD) of subjects at the baseline was 73.6 (5.9) years, and 46.2% of them were men. The proportions of the various K6 score groups were 65.2% for <5, 23.6% for 5–9, 6.5% for 10–12, and 4.8% for ≥13.

Table 1 shows the baseline characteristics of participants according to the K6 score groups. Participants with higher K6 scores were more likely to have a history of disease, and were less likely to be men, to have better motor function, and to have no lack of social support.

### 3.2. Psychological Distress and Incident Functional Disability

Table 2 shows the main results for the relationship between the K6 score and incident functional disability. Significantly higher risk was observed in the higher K6 score groups; the multiple-adjusted HRs (95% CIs) for incident functional disability were 1.14 (1.06–1.22) for 5–9 points, 1.28 (1.15–1.43) for 10–12 points, and 1.62 (1.44–1.84) for ≥13 points, in comparison with <5 points (*p*-trend < 0.001).

This inverse association did not differ significantly between the sexes (*p*-interaction = 0.651; Table 3).

We also obtained similar results even when the multiple imputation method was applied; the multiple-adjusted HRs (95% CIs) for incident functional disability were 1.14 (1.06–1.22) for 5–9 points, 1.23 (1.08–1.39) for 10–12 points, and 1.51 (1.34–1.70) for ≥13 points, in comparison with <5 points (*p*-trend < 0.001; table not shown).

### 3.3. Population Attributable Fraction

The PAF_ALL_ (PAF of K6 score ≥5) was 7.2% (95% CIs: 4.9–9.4%). The PAFs for each of the K6 score groups were 3.0% (95% CIs: 1.3–4.7%) for 5–9 points, 1.7% (95% CIs: 0.9–2.6%) for 10–12 points, and 2.6% (95% CIs: 1.8–3.3%) for ≥13 points.

The PAF_+1_ was 5.4% (95% CIs: 3.9–6.8%).

### 3.4. Sensitivity Analysis

We also conducted stratified analysis by age subgroup (Table 4). A dose–response relationship was observed irrespective of the age subgroup (*p*-interaction = 0.425).

To confirm whether there was a relationship between the K6 score and incident functional disability, irrespective of motor function, we also conducted stratified analysis (Table 5). A dose–response relationship was observed irrespective of motor function (*p*-interaction = 0.425).

Additionally, we also conducted stratified analysis by social support subgroup (Table 6), and observed a dose–response relationship irrespective of the subgroup (*p*-interaction = 0.986).

## 4. Discussion

In this cohort study, we observed a significant dose–response relationship between the score on a psychological distress scale (K6) and incident functional disability, and a significantly higher risk even for a mild level of psychological distress (K6 score ≥5). The lower risk of incident functional disability was consistent even after stratifying for motor function at the baseline.

The previous study of elderly disaster survivors also observed a significant dose–response relationship between the K6 (three categories; K6 score ≤9, 10–12, ≥13) and incident functional disability [10]. This previous study focused on a relatively higher level of psychological stress (K6 score ≥10), and did not examine mild levels of psychological distress (K6 score ≥5).

In the present study, any level of psychological distress (K6 score ≥5) had considerable impact on incident functional disability (PAF_ALL_ = 7.2%). However, the degree of impact was smaller than that in a previous cross-sectional study (39.3%) [12]. Because functional decline is known to be a risk factor for psychological distress, the previous result may have included such a reverse causality effect (i.e., this result of a cross-sectional study might indicate a reciprocal relationship between psychological distress and functional disability) [5]. Therefore, it may not be appropriate to simply compare values of PAF between the present study and the previous cross-sectional study.

Interestingly, the PAF for mild psychological distress (K6 score 5–9: 3.0%) was higher than that for severe psychological distress (K6 score ≥13: 2.6%). When psychological distress was summed as mild to moderate (K6 score 5–12: 4.7%), the PAF was approximately twice that for severe psychological distress (K6 score ≥13: 2.6%). This finding suggests that mild to moderate psychological distress should also be considered in any public health strategy for comprehensive prevention of functional disability.

Although the HR for severe psychological distress was highest among all the psychological distress levels, the prevalence of severe psychological distress was only one-fifth that of mild psychological distress. This relative difference in prevalence has also been observed in Japanese national statistics and world statistics [13,36]. Therefore, the impact of mild to moderate psychological distress would not have been unique to the present study population. Indeed, a previous study of adults (aged 18–64 years) also found that mild psychological distress had a considerable impact (in terms of PAF), and indicated that “mild psychological distress may be associated with more long-term disability than previously known” [11].

Our study had several strengths: (1) it was a large cohort study involving 12,365 persons; and (2) it had a follow-up rate of almost 100% (98.6%).

On the other hand, it also had some limitations. First, psychological distress was measured only once; we did not consider any changes in the participants’ psychological status. Second, we did not consider the causes of incident functional disability. Third, the target population was limited to residents in a rural Japanese community. Additional studies conducted in other settings will be needed to confirm the external validity of our findings. Fourth, not all potential confounding factors were considered. For example, we did not consider sensory function, living arrangement, or income.

## 5. Conclusions

Psychological distress, as measured by the K6, was associated with a significantly high risk of incident functional disability in Japanese elderly. Even a mild to moderate level of psychological distress had a considerable impact on incident functional disability.

## Figures and Tables

**Table 1:** Baseline characteristics according to K6 score (*n* = 12,365).

	Psychological Distress Scale (K6 Score)			
	<5	5–9	10–12	≥13
*n*	8064	2912	797	592
Age, mean (SD)	73.4 (5.9)	73.7 (5.9)	74.3 (6.2)	74.4 (6.1)
Men, %	48.3	43.9	41.4	35.1
History of disease, %				
Stroke	2.2	3.3	3.8	4.9
Hypertension	41.9	45.9	45.4	49.7
Myocardial infarction	4.1	6.3	7.2	7.4
Diabetes mellitus	11.0	13.4	13.3	15.2
Arthritis	13.4	19.4	22.1	23.8
Cancer	8.5	8.7	11.7	12.5
Better motor function, % ^1^	84.5	73.4	64.5	53.7
Smoking, %				
Current	13.6	13.2	13.3	13.0
Past	28.1	25.9	28.7	24.6
Never	58.3	60.9	58.0	62.5
Alcohol drinking, %				
Current	40.5	36.9	33.5	26.5
Past	10.4	11.5	14.5	15.6
Never	49.2	51.6	52.0	57.9
Body mass index (kg/m^2^), %				
<18.5	4.1	5.8	7.3	9.2
18.5–24.9	65.5	62.8	66.4	61.6
≥25	30.5	31.4	26.4	29.2
No lack of social support, %	80.4	71.8	66.9	55.4

^1^ Motor function score of the Kihon Checklist < 3.

**Table 2:** Association between K6 score and incident functional disability (*n* = 12,365).

	Psychological Distress Scale (K6 Score)				*p*-Trend
	<5	5–9	10–12	≥13	
Person-years	63,722	21,783	5498	3634	
Event, *n*	2720	1148	362	303	
Model 1 ^1^	1.00 (Reference)	1.26 (1.18–1.35)	1.55 (1.39–1.73)	2.01 (1.78–2.26)	<0.001
Model 2 ^2^	1.00 (Reference)	1.14 (1.06–1.22)	1.28 (1.15–1.43)	1.62 (1.44–1.84)	<0.001

^1^ Model 1 was adjusted for age and sex. ^2^ Model 2 was adjusted for age, sex, history of disease, motor function, smoking, alcohol drinking, body mass index, and social support.

**Table 3:** Association between K6 score and incident functional disability stratified by sex.

	Psychological Distress Scale (K6 Score)				*p*-Trend
	<5	5–9	10–12	≥13	
Men (*n* = 5709)					
Person-years	30,300	9594	2140	1250	
Event, *n*	1208	445	142	90	
Model 1 ^1^	1.00 (Reference)	1.23 (1.10–1.37)	1.74 (1.46–2.07)	1.83 (1.48–2.27)	<0.001
Model 2 ^2^	1.00 (Reference)	1.11 (0.99–1.24)	1.43 (1.20–1.71)	1.32 (1.05–1.65)	<0.001
Women (*n* = 6656)					
Person-years	33,422	12,189	3358	2384	
Event, *n*	1512	703	220	213	
Model 1 ^1^	1.00 (Reference)	1.28 (1.17–1.40)	1.45 (1.26–1.67)	2.09 (1.81–2.41)	<0.001
Model 2 ^2^	1.00 (Reference)	1.16 (1.06–1.27)	1.20 (1.04–1.39)	1.78 (1.53–2.06)	<0.001

^1^ Model 1 was adjusted for age (65–69, 70–74, 75–79, 80–84, or ≥85 years). ^2^ Model 2 was adjusted for age, history of disease, motor function, smoking, alcohol drinking, body mass index, and social support.

**Table 4:** Sensitivity analysis: stratified by age subgroup.

	Psychological Distress Scale (K6 Score)				*p*-Trend
	<5	5–9	10–12	≥13	
**<75 years old** (*n* = 7410)					
Person-years	42,949	14,812	3725	2429	
Event, *n*	974	418	149	117	
Model 1 ^1^	1.00 (Reference)	1.23 (1.09–1.37)	1.72 (1.45–2.05)	2.16 (1.79–2.62)	<0.001
Model 2 ^2^	1.00 (Reference)	1.10 (0.97–1.23)	1.39 (1.17–1.66)	1.55 (1.27–1.89)	<0.001
**≥75 years old** (*n* = 4955)					
Person-years	20,772	6971	1773	1205	
Event, *n*	1746	730	213	186	
Model 1 ^1^	1.00 (Reference)	1.28 (1.17–1.39)	1.45 (1.25–1.67)	1.92 (1.65–2.23)	<0.001
Model 2 ^2^	1.00 (Reference)	1.16 (1.07–1.27)	1.21 (1.04–1.40)	1.64 (1.40–1.91)	<0.001

^1^ Model 1 was adjusted for age (65–69 or 70–74 year in “<75 years old”; “75–79, 80–84, or ≥85 years” in “≥75 years old”) and sex. ^2^ Model 2 was adjusted for age, sex, history of disease, motor function, smoking, alcohol drinking, body mass index, and social support.

**Table 5:** Sensitivity analysis: stratified by motor function subgroup.

	Psychological Distress Scale (K6 Score)				*p*-Trend
	<5	5–9	10–12	≥13	
Lower motor function (*n* = 2457)					
Person-years	7444	4452	1517	1266	
Event, *n*	692	428	153	167	
Model 1 ^1^	1.00 (Reference)	1.10 (0.97–1.24)	1.18 (0.99–1.41)	1.69 (1.42–2.00)	<0.001
Model 2 ^2^	1.00 (Reference)	1.09 (0.97–1.23)	1.12 (0.94–1.34)	1.60 (1.34–1.91)	<0.001
Better motor function (*n* = 9354)					
Person-years	53,773	16,375	3638	2143	
Event, *n*	837	1887	647	184	
Model 1 ^1^	1.00 (Reference)	1.20 (1.10–1.32)	1.62 (1.39–1.88)	1.62 (1.34–1.95)	<0.001
Model 2 ^2^	1.00 (Reference)	1.15 (1.05–1.26)	1.48 (1.27–1.72)	1.49 (1.23–1.80)	<0.001

^1^ Model 1 was adjusted for age (65–69, 70–74, 75–79, 80–84, or ≥85 years) and sex. ^2^ Model 2 was adjusted for age, sex, history of disease, smoking, alcohol drinking, body mass index, and social support.

**Table 6:** Sensitivity analysis: stratified by social support subgroup.

	Psychological Distress Scale (K6 Score)				*p*-Trend
	<5	5–9	10–12	≥13	
**No lack** (*n* = 9326)					
Person-years	50,967	15,281	3594	1875	
Event, n	2132	840	238	165	
Model 1 ^1^	1.00 (Reference)	1.34 (1.23–1.45)	1.47 (1.28–1.68)	1.99 (1.70–2.33)	<0.001
Model 2 ^2^	1.00 (Reference)	1.22 (1.13–1.32)	1.21 (1.05–1.38)	1.67 (1.42–1.96)	<0.001
**Any lack** (*n* = 2893)					
Person-years	12,163	6179	1814	1678	
Event, n	545	296	120	131	
Model 1 ^1^	1.00 (Reference)	1.06 (0.92–1.22)	1.71 (1.40–2.09)	1.88 (1.55–2.27)	<0.001
Model 2 ^2^	1.00 (Reference)	0.97 (0.84–1.12)	1.46 (1.20–1.79)	1.58 (1.30–1.93)	<0.001

^1^ Model 1 was adjusted for age (65–69, 70–74, 75–79, 80–84, or ≥85 years) and sex. ^2^ Model 2 was adjusted for age, sex, history of disease, motor function, smoking, alcohol drinking, and body mass index.
